# Central and peripheral effects of ghrelin over the hypotension induced by endotoxemic shock

**DOI:** 10.1186/cc12993

**Published:** 2013-11-05

**Authors:** Felipe Faim, Marcelo Batalhão, Evelin Carnio

**Affiliations:** 1 Departamento de Fisiologia, Faculdade de Medicina de Ribeirão Preto, Universidade de São Paulo, Brazil; 2 Departamento de Enfermagem Geral e Especializada, Escola de Enfermagem de Ribeirão Preto, Universidade de São Paulo, Brazil

## Background

Since its discovery by Kojima and colleagues in 1999 [1], the hormone ghrelin has been studied in different contexts, since this peptide has the ability to promote hormonal, vascular and immune changes. His well-established functions are the release of growth hormone, by a mechanism distinct from the growth hormone release factor, and stimulation of hunger, by activating hippothalamic neurons, leading to release of neuropeptide Y, thus promoting orexigenic effects [2]. Because of its ability to release hormones, including vasopressin [3], and by possessing immunomodulatory properties, ghrelin has been studied in different contexts of inflammatory states, as present in endotoxemia and sepsis [4]. The infusion of lipopolysaccharide (LPS) is capable of generating an inflammatory state, with augmenting of TNFα, IL-1β and nitric oxide, which in turn leads to cardiac depression and systemic vasodilation and hypotension [5]. Due to its properties to modulate the inflammatory response, in a way of diminishing the levels of TNFα, IL-1β and nitric oxide, which are augmented in the endotoxemic state, as well the ability to augment the plasma levels of vasopressin, ghrelin emerges as a potential neuro-immunomodulator in hypotension caused by endotoxemia. We speculate that ghrelin, mediating the inflammatory response and by augmenting vasopressin blood levels, could attenuate the hypotension caused by endotoxin.

## Materials and methods

Male Wistar rats (250 to 300 g) had their jugular vein and/or their right cerebral ventricle cannulated for drug administration, and the femoral artery cannulated for mean arterial pressure (MAP) and heart rate (HR) records, respectively. All experimental procedures were approved by the Comitê de Ética em Experimentação Animal-campus de Ribeirão Preto (protocol number 12.1.1441.53.5). The endotoxemia model was induced by endovenous injection of lipopolysaccharide (LPS; 1.5 mg/kg). Data were compared using two-way analyses of variance and significant differences were obtained using the Bonferroni post test.

## Results

LPS administration leads to a drop in MAP in the first 2 hours, followed by a partial recovery of the MAP, and then a second drop in MAP, with a peak in 6 hours. The HR was augmented in this group. Systemic administration of ghrelin alone, through a bolus followed by subcutaneous implantation of an osmotic pump, did not alter the response, in comparison with the saline-treated group. The icv administration of ghrelin, however, diminished the HR in some intervals, although did not present a difference in MAP, in comparison with the saline-treated group. The administration of ghrelin, centrally and peripherally, when given at the same time as the LPS bolus, attenuated the first drop in MAP and completely restored the second drop present in the LPS group.

## Conclusions

Ghrelin is capable of attenuating the hypotension caused by endotoxin, and we speculate that the improvement is due to modulation of cytokines, nitric oxide and augmented vasopressin blood levels.
